# Case Report: Potential Role of Corticosteroids in the Management of Post-COVID-19 Pneumonia

**DOI:** 10.3389/fmed.2021.686806

**Published:** 2021-09-09

**Authors:** Houari Aissaoui, Anaïs Eskenazi, Valentin Suteau, Antoine Adenis, Kinan Drak Alsibai

**Affiliations:** ^1^Department of Medicine, Pulmonology Unit, Cayenne Hospital Center Andrée Rosemon, Cayenne, French Guiana; ^2^Department of Medicine, Cayenne Hospital Center Andrée Rosemon, Cayenne, French Guiana; ^3^Department of Pathology, Cayenne Hospital Center Andrée Rosemon, Cayenne, French Guiana; ^4^Centre d'Investigation Clinique Antilles-Guyane (Inserm 1424), Cayenne Hospital Center Andrée Rosemon, Cayenne, French Guiana; ^5^Center of Biological Resources (CRB Amazonie), Cayenne Hospital Center Andrée Rosemon, Cayenne, French Guiana

**Keywords:** severe COVID-19 pneumonia, ARDS, corticosteroid, post-COVID-19 infection, interstitial lung diseases, organizing pneumonia

## Abstract

Certain patients who recover from severe pneumonia due to coronavirus disease 2019 (COVID-19) remain symptomatic in the post-infectious period, either clinically, radiologically, or respiratory. The post-COVID-19 period is characterized by clinical symptoms of varying duration from one subject to another and does not seem to depend on the severity of initial pneumonia. The persisting inflammatory and/or immune reactions in the post-COVID-19 period may play a role in the development of pulmonary lesions. Here, we report the case of a 61-year-old man with severe COVID-19 pneumonia, complicated by acute respiratory distress syndrome and pulmonary embolism, which required the patient's admission to the intensive care unit and high-flow oxygen therapy. The patient was hospitalized for 23 days for the management of his severe COVID-19 pneumonia. Afterwards, he was discharged home following a negative SARS-CoV-2 PCR test. The post-COVID-19 period was characterized by a complex respiratory symptomatology associating cough, resting dyspnea, and exertional dyspnea requiring oxygen therapy for several weeks. Surprisingly, the follow-up chest CT scan performed 4 weeks after discharge revealed bilateral interstitial lung lesions. After ruling out pulmonary superinfection, the patient was treated with oral corticosteroid for 3 months at a digressive dose. In our case, the use of corticosteroid therapy in the post-COVID19 phase had improved the outcome of the lung disease. These benefits are characterized by a rapid symptomatic improvement, accelerated repair of pulmonary images, rapid oxygen withdrawal, and rapid return to daily activities.

## Introduction

The coronavirus disease 2019 (COVID-19) is a pandemic infectious disease caused by the novel coronavirus SARS-CoV-2 (Severe Acute Respiratory Syndrome coronavirus 2). In French Guiana, SARS-CoV-2 has infected 15,664 inhabitants and caused the death of 76 patients (according to the January 2021 epidemiological report of the National Health Agency of Guiana).

The final diagnosis is based on the detection of the SARS-CoV-2 virus and/or on the typical chest computed tomography (CT) scan findings of COVID-19 pneumonia including ground-glass opacities, and consolidation images that are mostly bilateral, subpleural, and peripheral (1, 2).

However, certain patients who recover from severe COVID-19 pneumonia remain symptomatic in the post-infectious period, either clinically, radiologically, or respiratory, despite a negative SARS-CoV-2 control test (3–5). The post-COVID-19 period is characterized by clinical symptoms of varying duration from one subject to another and does not seem to depend on the severity of initial pneumonia (6). The persistence of residual inflammation or immune response, and repair and remodeling mechanisms in the post-COVID-19 period may play a role in the development of new lung lesions or the aggravation of pre-existing lesions (7). In this article, we will discuss the potential role of corticosteroid therapy in the favorable course of post-COVID-19 pneumonia. The patient was treated for a severe form of COVID-19 pneumonia, but retained post-infectious respiratory symptoms, with radiological images in favor of rapidly progressive bilateral interstitial lung disease.

## Case Report

We report the case of a 61-year-old man, non-smoker, with a history of obesity, well-controlled intermittent asthma, and discrete bronchial dilatation (BD) of undetermined cause localized to the lingula, with a family history of BD.

**Figure 1:**
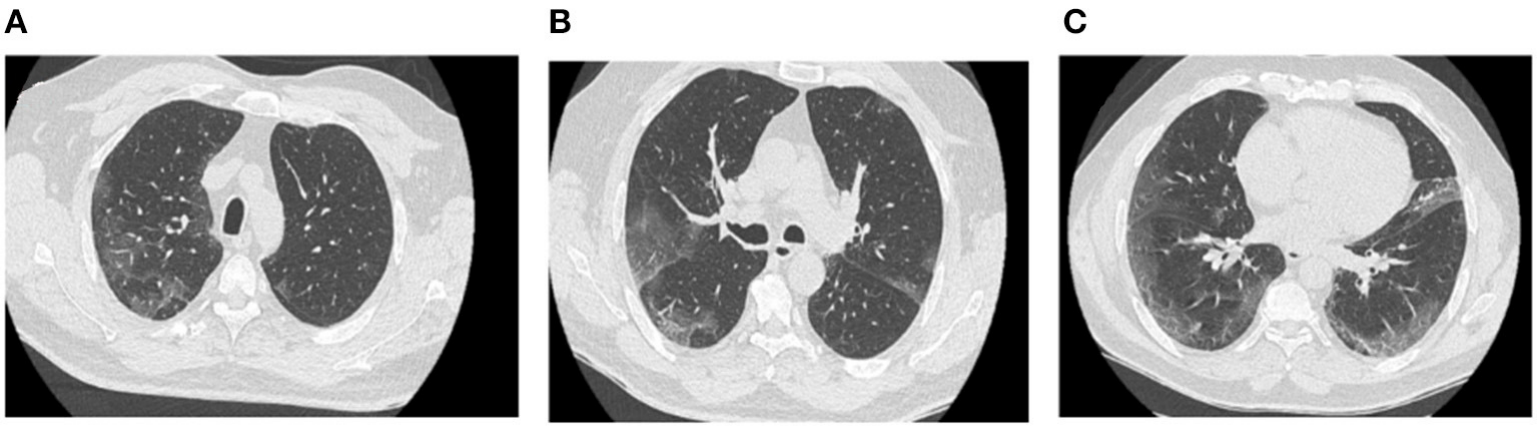
Chest CT scan of COVID-19 pneumonia (first chest CT scan). Axial sections of the chest CT scan at different levels show bilateral ground-glass opacities and consolidation images that are bilateral, subpleural, and located mainly in posterior regions **(A–C)**.

The patient consulted the emergency department of our hospital in June 2020 following the onset of dyspnea and diffuse sibilance. The symptoms had started 4 days earlier with fever, dry cough, chest pain, and diarrhea. The chest CT scan performed on the fifth day of symptom onset showed ground-glass opacities suggestive of COVID-19 with moderate lung involvement (Figure 1). This diagnosis was confirmed by a positive SARS-CoV-2 RT-PCR test.

Due to the mildness of symptoms at this stage, the patient was sent home for confinement. However, the patient returned to the emergency department 2 days later because of a worsening of his dyspnea. At this time, the desaturation was 89% on room air. Following this evolution, the patient was hospitalized in the COVID-19 unit. During the hospitalization, the patient had developed an acute respiratory distress syndrome (ARDS), characterized by a marked worsening of respiratory symptoms with an increase in oxygen requirements assessed at 6 L/min on the second day of hospitalization and up to 15 L/min the following day. Given this rapid deterioration, the patient was treated by a short-term corticosteroid with intravenous methylprednisolone 100 mg per day, and anticoagulant enoxaparin (0.4 ml) twice a day.

Three days later, the patient was transferred to the intensive care unit following the increase in polypnea and asthenia. Oxygen therapy by Optiflow with 100% fraction of inspired oxygen (FiO_2_) and 50 L/min airflows was prescribed for 6 days. The thoracic angioscanner showed a right posterior and basal segmental pulmonary embolism and a worsening of pulmonary involvement. Therefore, we introduced the anticoagulant enoxaparin at a curative dose of 6,000 U twice a day and a corticosteroid therapy by dexamethasone 6 mg per day for 10 days. As a result of this treatment, we noted a progressive clinical and respiratory improvement with a switch to 6 L/min oxygen. Thus, the patient was transferred back to the COVID-19 unit for monitoring. Nevertheless, he developed a stress and anxiety and refused oxygen therapy by systematically removing the oxygen cannula.

Following a marked improvement in symptoms and a negative SARS-CoV-2 RT-PCR control test, the patient was discharged home after 23 days of symptom onset with oxygen therapy at 6 L/min.

**Figure 2:**
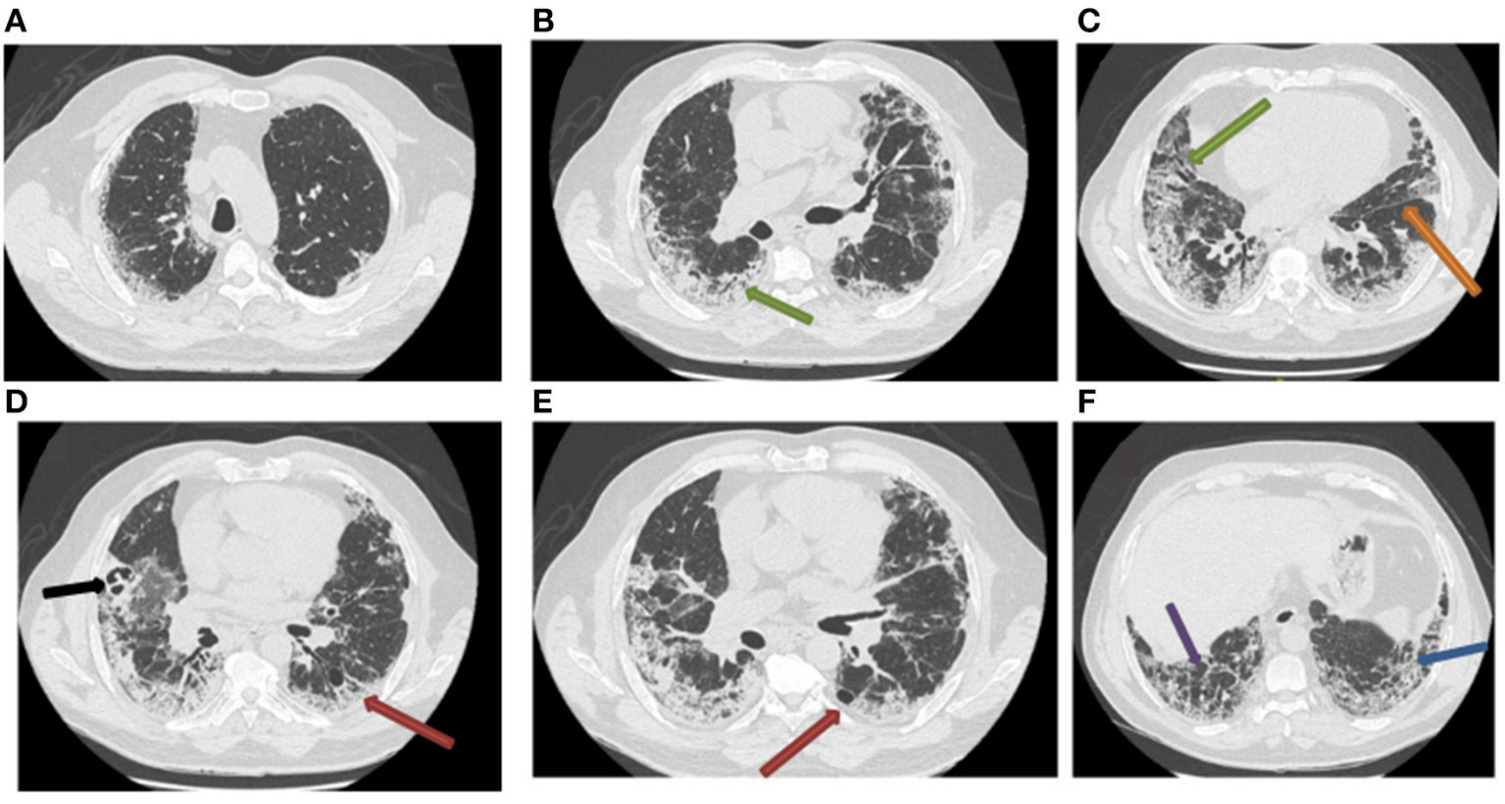
Follow-up chest CT scan of post-COVID-19 pneumonia (second chest CT scan). The chest CT scan performed 4 weeks after the management of severe COVID-19 pneumonia reveals the appearance of bronchiectasis (**A–C**; green arrow), subpleural reticulations (**F**; blue arrow), lobular distortions (**F**; violet arrow), fissure deformity (**C**; orange arrow), paracicatricial emphysema (**D,E**; red arrow), and cavitation in the right middle lobe (**D**; black arrow).

Two days later, the patient returned to the emergency department following the reappearance of new respiratory distress at the time of his morning toilet with the sensation of suffocation and dyspnea when talking. The respiratory rate was 45 beats/min with a saturation of 87% on 6 L/min oxygen. The respiratory functional exploration (RFE) showed a severe restrictive syndrome with a total lung capacity (TLC) of 39% of the predicted value. The chest CT scan performed 4 weeks after the onset of symptoms revealed a diffuse interstitial lung lesion developed on the previously observed COVID-19 images. The CT scan also showed reticulation and globular distortion, and traction bronchiectasis associated with ground-glass opacities and scissural deformation (Figures 2A–F). A paracicatricial emphysema without honeycomb signs and the appearance of a cavity image in the right middle lobe were also noted (Figures 2D,E).

Due to these new radiological images, and to eliminate a superinfection, a bronchoscopy with a bronchioalveolar lavage (BAL) was performed in the right middle lobe where the cavity image was located (Figure 2D). The cytological study showed a cellularity of 110,000 cells/ml, and inflammatory formula (macrophages 76%, lymphocytes 5%, neutrophils 15%, and eosinophils 4%), with few red blood cells. Bacteriological and mycological analyses of BAL by direct examination and culture did not show any pathogen. The result of the SARS-CoV-2 PCR test performed on the BAL was negative.

In view of this unusual radiological evolution suggestive of rapid-onset interstitial lung disease, corticotherapy by prednisolone (0.5 mg/kg/day) was started at the end of July 2020 with a gradual decrease in the dose, as follows: 50 mg/day for 1 month, 40 mg/day for 2 weeks, 30 mg/day for 2 weeks, 10 mg/day for 2 weeks, and finally 5 mg for 2 weeks.

The evolution was rapidly favorable after the first month of corticotherapy at 0.5 mg/kg with a decrease in oxygen requirements to 4 L/min and then to 2 L/min to complete weaning at the end of August 2020 (Table 1). Following motor physiotherapy instituted at home, the exertional dyspnea was gradually improved. The patient resumed his daily private and professional activities at the beginning of September 2020.

**Table 1:** Gradual and consistent improvement in gasometry under corticosteroid therapy from July to October 2020.

	**July 17**	**August 13**	**August 27**	**September 4**	**October 30**
pH (7.38–7.42)	7.45	7.43	7.41	7.42	7.42
PaO_2_ (75–100 mmHg)	52	61	71	74	73
PaCO_2_ (38–42 mmHg)	35	38	34	41	40
HCO_3_ (22–28 mEq/L)	24.3	25.2	21.6	26.6	25.9
SpO_2_ (94–100%)	90.0	94.1	96.0	96.9	95.3

*pH, arterial blood pH; PaO_2_, partial pressure of oxygen; PaCO_2_, partial pressure of carbon dioxide; SpO_2_, oxygen saturation; HCO_3_, bicarbonate*.

**Figure 3:**
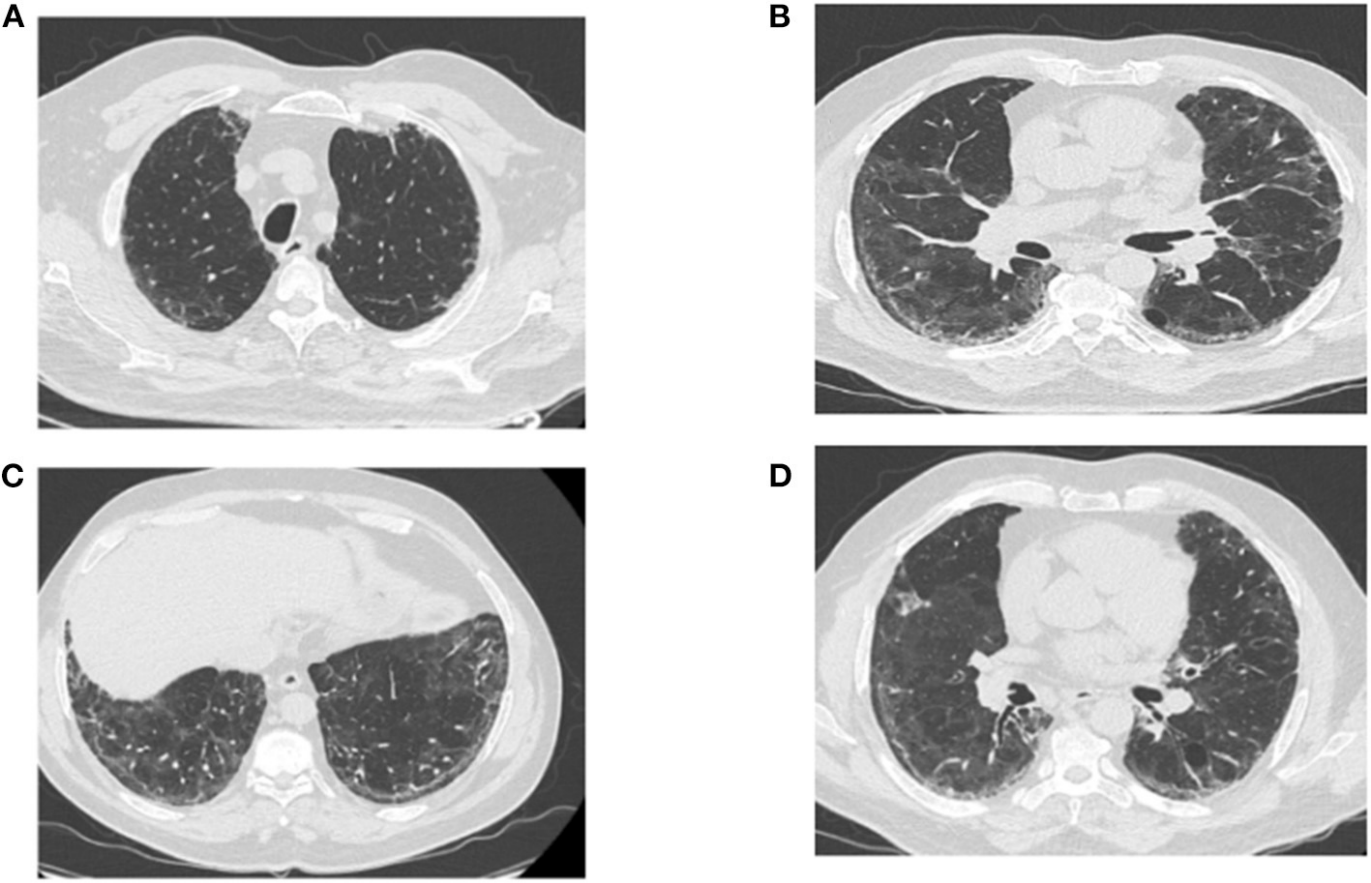
Post-corticotherapy chest CT scan (third chest CT scan). The chest CT scan performed after 3 months of corticotherapy of post-COVID-19 symptoms reveals the regression of ground-glass lesions and bronchiectasis, and the persistence of paracicatricial emphysema **(A–C)**. **(B–D)** Show the disappearance of the cavity image of the right middle lobe, the regression of retraction signs, and the persistence of few reticulation lesions.

The RFE at follow-up visits showed that TLC normalized at the fourth month of COVID-19 pneumonia onset, which corresponds to the third month of corticotherapy (Table 2). The blood analyses gradually improved and returned to normal by the end of October 2020 (Table 3). Surprisingly, the follow-up chest CT scan at the same period showed a significant and unexpected improvement of the pulmonary lesion characterized by the disappearance of bronchiectasis (Figures 3A–C), the significant reduction of reticulations, and the regression of retraction signs (Figures 3B–D). The CT scan also revealed the disappearance of the cavity image of the right middle lobe (Figure 3C), but the persistence of paracicatricial emphysema (Figures 3B,C).

**Table 2:** The normalization of respiratory function after 3 months of treatment with oral corticosteroids.

	**July 24**	**October 30**
TLC (%)	39	89.7
RV/TLC (%)	79.7	129.6
FVC (L)	1.78	2.81
FVC (%)	46	72.7
FEV_1_ (L)	1.37	2.30
FEV_1_ (%)	44.8	75.5
FEV_1_/FVC (%)	76.83	82

*FEV_1_, forced expiratory volume in 1 s; FVC, forced vital capacity; TLC, total lung capacity; RV, residual volume*.

**Table 3:** Blood analyses that gradually improved and returned to normal by the end of October 2020.

	**July 17**	**September 4**	**October 24**
Hemoglobin (13–18 g/dl)	12.7	12.9	13.5
Platelets (150–400 g/L)	243	209	281
Fibrinogen (2.38–4.98 g/L)	4.38	–	2.65
C reactive protein (<5 mg/L)	41	0.8	0.5
Ferritin (20–200 μg/L)	429	–	220
Phosphatase alkaline (40–130 U/L)	85	52	63
D Dimer (<500 ng/ml)	6,206	2,417	510
Lactic acid dehydrogenase (135–225 U/L)	304	288	269

## Discussion

In our patient, the pulmonary involvement related to SARS-CoV-2 was extensive and severe, complicated by ARDS and pulmonary embolism, which required to be managed by the intensive care unit with high-flow oxygen therapy.

The post-COVID-19 period was characterized by a complex respiratory symptomatology associating cough, resting dyspnea, and exertional dyspnea requiring oxygen therapy for several weeks, altering the patient's quality of life. The patient also developed stress and anxiety. These symptoms were associated with the development of a diffuse, severe, and rapidly progressive interstitial lung disease with signs of retraction, distortion, cavitation, and diffuse bronchiectasis. This post-COVID-19 pulmonary disease was of rapid onset, as it occurred within 4 weeks after the first symptoms.

Previous studies of SARS-CoV-2 infection have shown that the lung damage observed during the initial phase of COVID-19 pneumonia is the consequence of the host's inflammatory and immune response to the viral infection. On the tissue level, this inflammatory response can cause diffuse lung injury with the formation of hyaline membranes, fibrin exudates, epithelial damage, and diffuse hyperplasia of type II pneumocytes (8). Further analyses revealed that the intra-alveolar and interstitial fibrin deposits and chronic inflammatory infiltrate may appear a few weeks after the diagnosis of COVID-19 (9, 10). In this context, it can be assumed that the extreme immune response persists in post-SARS-CoV-2 infection and continues to evolve for several weeks, resulting in the maintenance of a state of chronic inflammation and ongoing lung damage.

In our case, the follow-up chest CT scan performed 4 weeks after discharge revealed bilateral interstitial lung lesions, suggesting unusual rapid-onset pulmonary fibrosis-like lesion. In addition, the CT scan revealed vascular congestion developed over ground-glass opacities (Figures 1A–C), associated with markedly dilated vessels (Figures 2B,E), suggesting microthrombosis lesions. These fibrosis-like lesions (11) and pulmonary vascular changes (12) related to COVID-19 have been described recently.

Previous studies have suggested that some COVID-19 patients can develop secondary organizing pneumonia (OP) (13–15). OP is defined as an intra-alveolar organized exudate composed of fibroblasts and myofibroblasts (16). The incidence of OP was 12.5% in a German cohort of patients with severe COVID-19 (13) and 4% in a more recent prospective observational study (14). The viral infections are the most common etiologies of secondary OP (17). The final diagnosis of OP requires histological assessment by a biopsy (18).

Myall et al. used radiological pattern to define OP as bilateral subpleural ground-glass infiltrates associated with subpleural and peribronchial linear dense consolidation, and traction bronchiectasis. The patients were offered treatment with corticosteroid and had improvements in lung function and chest imaging (14).

Our patient had a radiological pattern of OP with a patchy consolidation in peripheral and subpleural distribution, associated with subpleural reticulations and traction bronchiectasis (Figures 2B,D,E). In addition, there was paracicatricial emphysema and cavitation. In our case, confirmation by biopsy was not indicated due to the patient's condition.

The presence of a cavitation in COVID-19 pneumonia has been described recently (19). In the presence of such a lesion, superinfection must be ruled out. In our case, the results of the cytological, bacteriological, and mycological analyses of BAL were negative.

The effects of corticosteroid therapy in reducing mortality in critical and severe cases of COVID-19 pneumonia have been well-demonstrated, with an intermediate level of evidence (20).

**Figure 4:**
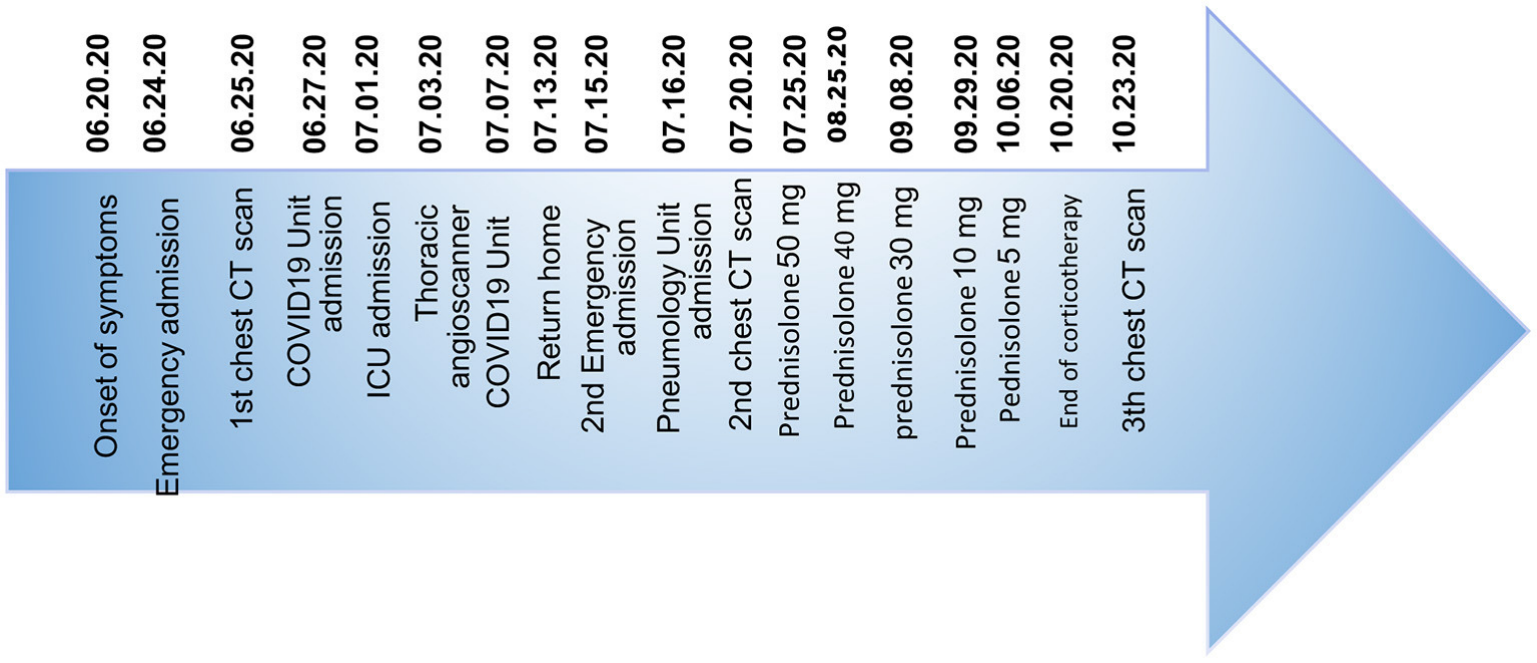
This timeline shows the patient's clinical history from the onset of COVID-19 pneumonia-related symptoms to the favorable evolution of post-COVID-19 symptoms and pulmonary lesions after corticosteroid therapy. The patient was discharged from the hospital on July 30, 2020 and completed his corticosteroid therapy at home until October 20, 2020.

In this case report, the use of corticosteroid therapy in post-COVID-19 allowed an acceleration of the repair of lung lesions very probably due to its anti-inflammatory effects. We even observed a reversibility of radiological lesions initially taken for signs of pulmonary fibrosis. The corticosteroid therapy allowed also a rapid recovery and normalization of respiratory function, as well as a normalization of the partial pressure of oxygen (PaO_2_) and a complete weaning of oxygen after only 1 month of treatment (Figure 4, Table 1).

## Conclusion

Here, we report a case of severe SARS-CoV-2 pulmonary lesion complicated by acute respiratory distress syndrome and pulmonary embolism, which required intensive care unit admission and high-flow oxygen therapy. The use of corticosteroid therapy in our patient improved the outcome of the pulmonary disease. We observed a rapid improvement of symptoms, accelerating repair of radiological images, rapid weaning from oxygen, and improved exercise tolerance with rapid return to daily activities.

The robust responses of corticosteroid therapy in our case presenting a radiological pattern of OP allowed the patient to return to his baseline clinical condition. Therefore, we believe that the use of corticosteroids is beneficial in survivors of severe COVID-19 pneumonia, who remain symptomatic in the post-infection period, and who present radiological features consistent with OP. Nevertheless, the proof of efficacy of such a treatment requires further validation by rigorously conducted randomized trials, codifying the dosage and duration of treatment.

## Data Availability Statement

The original contributions presented in the study are included in the article/supplementary material, further inquiries can be directed to the corresponding author.

## Ethics Statement

Written informed consent was obtained from the individual(s) for the publication of any potentially identifiable images or data included in this article. All the patients of our institute are informed that their biological samples and associated data may be used for scientific purposes, and have the right to object. An information leaflet is also given to the patient, in whom the information is reminded, and the procedure for withdrawing consent is described according to the European General Data Protection Regulation (GDPR 2018).

## Author Contributions

HA and KD: conceptualization, writing—original draft preparation, and supervision. HA, AE, VS, AA, and KD: investigation. HA, VS, and KD: resources. HA, AA, and KD: writing—review and editing. All authors contributed to the article and approved the submitted version.

## Conflict of Interest

The authors declare that the research was conducted in the absence of any commercial or financial relationships that could be construed as a potential conflict of interest.

## Publisher's Note

All claims expressed in this article are solely those of the authors and do not necessarily represent those of their affiliated organizations, or those of the publisher, the editors and the reviewers. Any product that may be evaluated in this article, or claim that may be made by its manufacturer, is not guaranteed or endorsed by the publisher.
